# A compartmentalization-free microfluidic digital assay for detecting picogram levels of protein analytes†

**DOI:** 10.1039/d5lc00103j

**Published:** 2025-05-12

**Authors:** Nguyen H. Le, N. Sathishkumar, Alinaghi Salari, Ryan Manning, Raymond E. Meyer, Cheuk W. Kan, Alexander D. Wiener, Martin A. Rossotti, Sheldon Decombe, Richard P. S. de Campos, M. Dean Chamberlain, Jamshid Tanha, Nira R. Pollock, David C. Duffy, Aaron R. Wheeler

**Affiliations:** a Department of Chemistry, University of Toronto 80 St. George Street Toronto ON M5S 3H6 Canada aaron.wheeler@utoronto.ca; b Terrence Donnelly Centre for Cellular and Biomolecular Research, University of Toronto 160 College Street Toronto ON M5S 3E1 Canada; c Quanterix Corporation 900 Middlesex Turnpike Billerica MA 01821 USA; d Human Health Therapeutics Research Centre, Life Sciences Division, National Research Council Canada 100 Sussex Drive Ottawa ON K1A 0R6 Canada; e Institute of Biomedical Engineering, University of Toronto 164 College Street Toronto ON M5S 3E2 Canada; f Department of Biochemistry, Microbiology and Immunology, Faculty of Medicine, University of Ottawa 451 Smyth Road Ottawa ON K1H 8M5 Canada; g Department of Laboratory Medicine, Boston Children's Hospital 300 Longwood Avenue Boston MA 02115 USA

## Abstract

Digitalizing the signals generated from single protein molecules has significantly improved the sensitivity of immunoassays compared to traditional analog “bulk” measurements. The single molecule array (Simoa) technology, for instance, leverages counting of single molecules on magnetic beads to detect low-abundance proteins in biofluids. While existing digital detection platforms are ultra-sensitive, they typically require compartmentalization and complex and bulky analysis equipment, limiting their applicability in resource-limited settings. Here, we introduce a compartmentalization-free digital detection technique, that allows for much more straightforward detection analysis. We applied this method to a model assay for detecting the SARS-CoV-2 spike protein and compared its performance to alternative techniques. We optimized the new method for digital microfluidics and present preliminary results using an automated system to analyze undiluted human saliva samples, with imaging performed on a portable optical system. We propose that future iterations of the scheme introduced here have the potential to enable a wide range of applications beyond the laboratory.

## Abstract 2

This work introduces a compartmentalization-free digital detection technique that enhances sensitivity for detecting low-abundance proteins in an automated microfluidic format.

## Graphical abstract

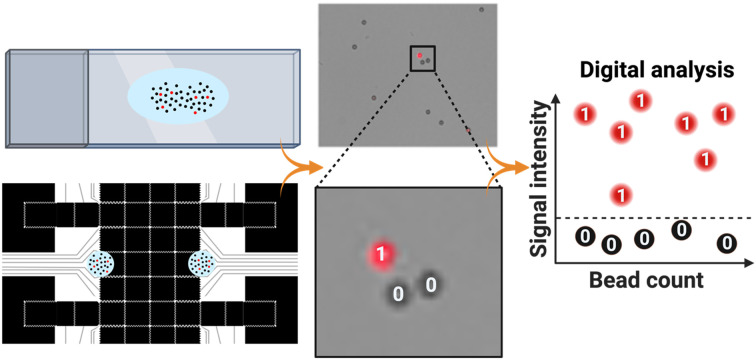

## Introduction

Sensitive methods for molecular analysis play a pivotal role in numerous applications, ranging from medical diagnostics to environmental sciences. In conventional analog detection schemes, the concentration of target molecules is directly correlated with the intensity of the signal obtained from the bulk sample. In digital detection, however, the quantification of molecules is determined by partitioning the liquid sample into discrete compartments and counting positive events. Compartments exhibiting analytical signals (typically from fluorescent reporters) above background levels are considered “1”s or “on”, while the others are categorized as “0”s or “off”. Leveraging Poisson statistics, one can precisely determine the analyte concentration by counting the “on” *versus* “off” compartments.^1^ Various compartmentalization strategies, ranging from femtoliter-sized wells^2,3^ and chambers^4^ to droplets^5–8^ have been developed for digital assays of proteins and cells, each offering advantages and drawbacks. For protein analytes, the gold standard for digital detection has been the single-molecule array (Simoa) technology that was developed by Quanterix Corporation. In Simoa, protein molecules are captured on antibody-coated magnetic beads, labeled with enzymes, and individual beads are compartmentalized into sealed, femtoliter microwells.^9^ As with other digital detection techniques, the partitioning mechanism (in this case, the sealed microwells) serves to confine the soluble fluorescent reporters in small (∼50 fL) volumes, so that the “on” and “off” signals can be readily distinguished from each other. The state-of-the-art for digital protein detection was recently reviewed in an accessible, comprehensive tutorial review article.^10^

While Simoa has set the standard for digital protein detection, the necessity of loading and sealing of beads and enzyme substrate into arrays of microwells creates complexities in fluid handling and consumable design. Likewise, the analytical platforms that have been developed to read these arrays, such as the Quanterix HD-X immunoassay analyzer,^11^ rely on sophisticated and bulky instrumentation for sample preparation, assay execution, signal readout and data analysis. These types of systems are well-suited to centralized clinical laboratories but limit the availability of digital protein detection in locations outside of the laboratory, where biomarker detection demands a more accessible solution. One option to help reduce the complexities of digital detection of proteins is to read digital signals from randomly distributed beads on a surface (*i.e.*, “compartmentalization-free”), rather than from beads sealed in microwells. This approach can only work for a label that is localized on the beads such that it cannot diffuse away from the bead. This idea was first demonstrated in 2016,^12^ and fully realized in three reports^13–15^ in 2020–2023. In the first two reports, Maley *et al.*^13^ and Ito *et al.*^14^ used tyramide signal amplification (TSA) to localize the signal to the beads, while in the third report, Wu *et al.*^15^ used rolling circle amplification (RCA) to localize the signal to the beads. Our work here was inspired by these initial seminal reports.

The aforementioned reports^13–15^ of compartmentalization-free digital protein detection share a powerful group of characteristics that we call “DABBS” – that is, they are designed for d̲igital detection and rely on molecular a̲mplification for high sensitivity, with the fluorescent reporters being b̲ead-b̲ound (on randomly distributed beads) in a s̲tatic field of view. However, in each of these previous methods,^13–15^ the DABBS analyses were implemented manually (with multiple pipetting steps), and they were not integrated into a format that is appropriate for portable, automated applications outside of the laboratory. Here, we have addressed this limitation, introducing a model DABBS assay designed to be implemented by digital microfluidics (DMF).

DMF is a good fit for the challenge of automating DABBS detection, as it provides for integrated fluid manipulation^16,17^ in a format that has been validated for magnetic-bead-based applications in remote settings.^18,19^ Here, we developed a new DABBS scheme relying on RCA on magnetic beads, and evaluated its performance relative to previous DABBS techniques. As a proof-of-concept, SARS-CoV-2 spike protein was selected as target antigen for DABBS assay development. The performance of the method was compared with alternative DABBS and non-DABBS techniques. Then the method was optimized and adapted to DMF, and its utility was assessed using an automated system to analyze undiluted human saliva samples, with imaging performed by a portable optical system. Based on the data shown here, we propose that future iterations of the microfluidic DABBS scheme have the potential to enable a wide range of diagnostic applications beyond the laboratory.

## Experimental

### Reagents and materials

Dynabeads™ M-270 Epoxy, Dynabeads™ M-280 Streptavidin, and Dynabeads™ Antibody Coupling Kit were purchased from Thermo Fisher Scientific (ON, Canada). Tetronic 90R4 was generously donated by BASF Corporation (BASF Corp., Germany). T4 DNA ligase and phi29 DNA polymerase were purchased from New England Biolabs (MA, USA). 3,3′,5,5′-tetramethylbenzidine (TMB) was purchased from Thermo Fischer Scientific (ON, Canada). All other reagents, including Tris-buffered saline with 0.1% v/v Tween® 20 detergent (TBST), SuperBlock™ in phosphate-buffered saline (PBS), SuperBlock™ in Tris-buffered saline (TBS), 1× Tris–EDTA (TE) buffer, horseradish peroxidase (HRP)-conjugated streptavidin, and streptavidin–Cy5 conjugate, were obtained from Sigma Aldrich (ON, Canada). Chromium and photoresist-coated glass slides (3′′ × 3′′) used to fabricate DMF devices were purchased from Telic Company (CA, USA). ITO-coated glass slides (25 mm × 75 mm × 0.7 mm) were sourced from Riley Supplies (ON, Canada). Parylene-C dimer was supplied by Specialty Coating System (IN, USA). FluoroPel 1101 V and PFC110 solvent were purchased from Cytonix, LLC (MD, USA). Oligonucleotides, DNA template, biotin-modified DNA primer, and Cy5-fluorescent DNA probe were obtained from Integrated DNA Technologies (IA, USA). Deoxyribonucleotide triphosphates (dNTPs) were purchased from Invitrogen (CA, USA). Streptavidin-modified DNA primer was purchased from BioSynthesis (TX, USA). The DNA template (phosphorylated on the 5′ end) was 5′-GCG TCT TGT AGT TCC CGT CCT GCT CCA CGA TGG TGT ACT GCT CCA CGA TGG TGT ACT GCT CCA CGA TGG TGT AAA CTT GAC TTC AGC AC-3′, the biotin-primer (biotinylated on the 5′ end) was 5′-GAC GGG AAC TAC AAG ACG CGT GCT GAA GTC AAG TT-3′, the streptavidin-primer (modified with streptavidin on the 5′ end) was 5′-GAC GGG AAC TAC AAG ACG CGT GCT GAA GTC AAG TT-3′, and the fluorescent probe (modified with Cy5 on the 5′ end) was 5′-CTG CTC CAC GAT GGT GTA-3′. A recombinant trimeric SARS-CoV-2 spike glycoprotein^20,21^ (Reference Material SMT1-1, Wuhan; molecular weight 551 kDa), referenced here as “antigen,” was provided by the National Research Council Canada. A panel of camelid antigen binding fragment of heavy-chain-only antibodies (V_H_Hs) specific to SMT1-1 were fused to human IgG Fc region and/or were site-specifically conjugated to biotin in-house, and were extensively characterized as described previously.^22^ A pair of these reagents, referenced here as “capture nanobody” (V_H_H 11-Fc) and a biotinylated “detection nanobody” (V_H_H 1d-biotin) was selected and their stability and antigen binding affinity was characterized in saliva, as described elsewhere.^23^

### Fabrication and operation of digital microfluidic devices

“Assay” (A) and “Image” (I) devices were formed (Fig. S1†). For both designs, DMF bottom plates were formed from chromium-coated glass substrates at the Centre for Research and Applications in Fluidic Technologies (CRAFT), following previously described methods^24,25^ involving UV photolithography and wet etching. Patterned bottom plates were then coated with a ∼6 μm layer of parylene-C through chemical vapor deposition at the Toronto Nanofabrication Center (TNFC), followed by spin coating with a 1% w/w solution of FluoroPel PFC 1101 V dissolved in PFC110 at 2000 rpm for 30 s. The coated bottom plates were baked in a dry oven at 110 °C for 15 min. Each type-A bottom plate featured an array of 91 standard electrodes (each with a square body of 2.0 mm × 2.0 mm and ∼1.0 mm × 0.4 mm extruded legs on each side) connected to 10 rectangular reservoir electrodes (6.5 mm × 12.0 mm) *via* 10 dispensing electrodes (each with a rectangular body of 2.0 mm × 4.5 mm and ∼1.0 mm × 0.4 mm extruded legs on the shorter side), with inter-electrode gaps of 100 μm on a 3′′ × 3′′ substrate. In some experiments, the type-A design was modified to include an array of five round densifying electrodes (each with a diameter of 1 mm, spaced 12.5 mm from each other), each positioned between neighboring standard electrodes. Type-I bottom plates featured a similar design, modified to include two round imaging windows (chromium free, 2 mm diameter, 8.8 mm from each other), positioned symmetrically at the outer edges of two standard-sized electrodes on a 2′′ × 3′′ substrate. DMF top plates used with both types of devices were prepared by dip-coating 1′′ × 3′′ ITO-coated glass slides in FluoroPel solution (1% w/w PFC 1101 V in PFC110) and then heating them in a dry oven at 110 °C for 15 min. Top plates used with type-I devices were further modified to remove the hydrophobic layer by micromilling in two round hydrophilic regions (2 mm diameter, spaced 8.8 mm apart) to match the imaging windows on the bottom plate. Devices were assembled by sandwiching top and bottom plates with spacers formed from two layers of double-sided tape (3 M Co., MN, USA) with a thickness of approximately 180 μm (making sure to align the bottom-plate windows and top-plate hydrophilic regions in type-I devices). A piece of conductive copper tape (3M Co., MN, USA, 180 μm thick) was placed onto the ground electrodes connecting the top and bottom plate. The volume of a unit droplet on a type-A device, defined as a droplet that covers a standard electrode, was approximately 1 μL. Devices were interfaced through pogo pin connectors to a custom version of Dropbot^26^ bearing a movable magnetic lens described in detail elsewhere.^19^ Droplets were actuated by applying a force of 25 μN mm^−1^ using the open-source MicroDrop 3.0 software, under conditions determined to be below the saturation force^27^ for all the liquids used.

### DABBS for streptavidin–biotin binding assays

An RCA protocol was developed for reporting streptavidin–biotin binding on beads, building from methods reported previously.^28–31^ Briefly, DNA template and biotin-primer were separately diluted in deionized water to the desired concentration. 2 μL of DNA template was added to 6 μL of biotin-primer at a molar ratio of 1 : 1. 4 μL of ligation solution [containing 0.15 M KCl, 2 mg mL^−1^ of non-bovine derived recombinant albumin, 2× T4 DNA ligase buffer, and 2 U mL^−1^ of T4 DNA ligase (400 000 U mL^−1^)], was prepared in PCR tubes. The tubes were then incubated at 30 °C for 30 min. 2 μL of an aqueous suspension of streptavidin-coated paramagnetic beads (1.3 × 10^6^ Dynabeads™ M-280 streptavidin beads) was added to the mixture and mixed using a rotator for 30 min to facilitate biotin–streptavidin interaction. The beads were subsequently washed three times by pelleting using a magnetic rack and resuspending in equivalent volumes of 1× TBST. After washing, 20 μL of RCA mixture, containing 1× phi29 reaction buffer, 2.5 U phi29 DNA polymerase, 1 mg mL^−1^ non-bovine derived recombinant albumin, and 0.14 mM deoxynucleotide mix, was added to the pelletized beads. The tubes were then incubated on a rotator for 1 h at room temperature. Following incubation, the RCA mix was aspirated after pelleting the magnetic beads using a magnetic rack. Subsequently, the beads were resuspended in 20 μL of 0.1 μM Cy5-labelled DNA probe in a buffer containing SuperBlock™ TBS, TE buffer (1 : 1 ratio) and 0.3 M NaCl. The tubes were then incubated at 50 °C, 40 °C, and 30 °C for 10 min each in an incubator. Finally, the aliquots of bead suspension were transferred onto a microscope slide for imaging, as described below in the “Imaging” section.

### DABBS for SARS-CoV-2 spike protein assays

#### Preparation of capture-nanobody-coated magnetic beads

Dynabead™ M-270 Epoxy beads were coated with capture nanobody using the Dynabeads Antibody Coupling Kit (Thermo Fisher Scientific, ON, Canada) following manufacturer instructions. Briefly, 3 mg of magnetic beads was dispersed in 1 mL of the kit's C1 buffer and then pelleted, disposing of the supernatant. 3.75 μL of an aqueous solution of capture nanobody (4 mg mL^−1^) was diluted in 146.25 μL of the kit's C1 buffer and then added to the suspended magnetic beads referenced above to achieve a final concentration of 5 μg of nanobody per milligram of beads. 150 μL of the kit's C2 buffer was added to the mixture and incubated at 37 °C for 16–24 h without allowing the beads to settle using Roto-Mini™ Plus Rotator (Benchmark, NJ, USA). After incubation, the beads were washed by pelleting and resuspending in the kit's wash buffers HB, LB, and SB (sequentially). Finally, the 3 mg of beads were suspended in 300 μL of the kit's C2 buffer and stored at 4 °C until use.

#### Ligation of DNA template on streptavidin-primer

A solution of DNA template (100 μM) was initially annealed at 95 °C for 2 min and then allowed to cool to room temperature. Subsequently, 20 μL of the DNA template solution was combined with 20 μL of an 84 μM streptavidin-primer solution, 6 μL of 10× ligation reaction buffer (New England Biolabs, MA, USA), and 16.5 μL of T4 DNA ligase (400 000 U mL^−1^). The mixture was incubated on a rotator overnight at room temperature. The resulting streptavidin-primer-template (SPT) products at 18.7 μM were stored in aliquots at −20 °C until use.

#### Assay preparation

Prior to running assays, detection nanobody and SPT were diluted in SuperBlock™ PBS to their optimal concentrations, as described in Note S1, Fig. S2 and S3.† Recombinant spike protein (antigen) was also diluted into SuperBlock™ PBS or in pooled saliva from human patient samples (MyBioResource, CA, USA) at various concentrations. Capture nanobody-modified magnetic particle suspensions were diluted to the densities indicated below for off-chip (in centrifuge tubes) or on-chip (on DMF devices) experiments. Finally, all reagents and samples used for on-chip experiments were supplemented with 0.01% w/v surfactant Tetronic 90R4.

#### Off-chip fluorometric DABBS assay procedure

A 2 μL aliquot of capture nanobody-coated bead suspension (1.3 × 10^6^ beads in the SB buffer of the antibody coupling kit) was used for each assay in a PCR tube. The beads were initially blocked and washed by pelleting using a magnetic rack and resuspending in an equivalent volume of SuperBlock™ PBS and incubating for 10 min at room temperature. Subsequently, a 100 μL aliquot of antigen and a 10 μL aliquot of biotinylated detection nanobody were added to the bead suspension. The sample was then mixed using a rotator for 1 h at room temperature to allow for the interaction, and the beads were then washed three times in equivalent volumes of SuperBlock™ PBS using a magnetic rack. A 100 μL aliquot of 27 nM SPT in SuperBlock™ PBS was added to the bead suspension. The mixture was incubated for 30 min at room temperature and then washed six times in equivalent volumes of SuperBlock™ PBS using a magnetic rack. After washing, the RCA reaction and Cy5-DNA labeling were performed, and the beads transferred to a microscope slide for imaging as described below.

#### On-chip fluorometric DABBS assay procedure

A custom, 19-step protocol was developed for DABBS assays on type-A devices. (1) A double-unit droplet of capture nanobody-coated magnetic bead suspension (containing various numbers but typically 2.0 × 10^5^ beads) was dispensed from a reservoir, and the beads were immobilized on the surface by engaging a magnetic lens,^18,19^ and the supernatant was moved to waste. (2) The magnetic lens was disengaged, and a double-unit droplet of the sample was dispensed and delivered to resuspend the beads. The mixture was supplemented with a freshly dispensed double-unit droplet of detection nanobody solution, and the combined droplet was moved in a circular path continuously for 1 h at room temperature. The magnetic lens was engaged to immobilize the beads, and the supernatant droplet was moved to waste. (3–8) The beads were washed six times, in each case disengaging and engaging the magnetic lens to (i) resuspend them in a freshly dispensed double-unit droplet of wash buffer and (ii) immobilize them to move the supernatant to waste. (9) The magnetic lens was disengaged, and the beads were resuspended in a freshly dispensed double-unit droplet of SPT solution, which was moved in a circular path continuously for 15 min at room temperature. The magnetic lens was engaged to immobilize the beads, and the supernatant droplet was moved to waste. (10–15) The beads were washed six times, repeating steps 3–8. (16) The magnetic lens was disengaged, and the beads were resuspended in a freshly dispensed double-unit droplet of RCA reagent mixture, which was moved in a circular path continuously for 1 h at room temperature. The magnetic lens was engaged to immobilize the beads, and the supernatant droplet was moved to waste. (17) The beads were washed once, repeating step 3. (18) The magnetic lens was disengaged, and the beads were resuspended in a freshly dispensed quintuple-unit droplet of 0.1 μM Cy5-labeled DNA probe solution. The device was incubated for 10 min at 50 °C, followed by 10 min at 30 °C. (19) A triple-unit droplet of SuperBlock™ TBS was dispensed and merged with the bead suspension to increase the total volume to ∼8 μL, which was moved to the reservoir electrode, for collection by pipette for imaging off-chip or on-chip (both procedures detailed below). Typically, steps 1–19 of the procedure were applied to five conditions in parallel (*e.g.*, four different samples and a negative control, in which sample was substituted with the wash buffer).

#### Off-chip non-DABBS assay procedures

A series of related-but-non-DABBS assays for SARS-CoV-2 spike protein were carried out off-chip. They included digital and analog fluorometric assays without amplification described in Note S2† (Fig. S4†), and an analog colorimetric assay with amplification described in Note S3† (Fig. S5†).

### Imaging

For off-chip imaging, bead suspensions collected after experiments were gently mixed by pipetting up and down several times. A ∼2.5 μL aliquot of each suspension was then pipetted onto a microscope slide. A clean coverslip was placed on top to enclose the beads, which were allowed to settle for 1 min. Brightfield and fluorescence images of beads were captured using an upright Nikon Eclipse (Ni-e) microscope equipped with an SCMOS camera, a 20× objective, and an X-cite XYLIS light source. Brightfield images were acquired with an exposure time of 200 μs, while epifluorescence images were obtained using a Cy5 filter cube (excitation filter: 590–650 nm; dichroic mirror: 660 nm; emission filter: 662.5–737.5 nm) with a 1 s exposure time. Both brightfield and fluorescence images were acquired for each frame, and multiple frames were obtained to be able to image (on average) ∼21% of beads. Briefly, approximately 100–150 frames of 20× magnification images were acquired for each sample using an automated motorized stage controlled by NIS-Element software, resulting in an average total imaging time of approximately 15–30 min. For on-chip imaging, for logistical reasons, bead suspensions collected after processing on type-A devices were shipped to a separate site, then washed twice in equal volumes of a buffer comprising SuperBlock™ TBS and TE buffer in a 1 : 1 ratio supplemented with 0.3 M NaCl, before resuspending in 10 μL of the same buffer. A 2 μL aliquot of this suspension was loaded onto a type-I DMF device, moved continuously for 1 min, delivered to the imaging windows, and then imaged using a custom-built, portable microscope, described in detail elsewhere.^32^ Briefly, the portable microscope was equipped with a red LED (Lumileds, CA, USA), an excitation filter (Alluxa, CA, USA), a bifurcated fiberoptic lightguide (low fluorescence) (Fiberoptic Systems, CA, USA), two machine vision lenses that together produce 2× magnification (Edmund Scientific, NJ, USA, and Navitar, NY, USA), a dual-band emission filter (IDEX/Semrock, NY, USA), and a CMOS camera (Basler, Germany). First, darkfield images were captured with an exposure time of 50 ms followed by fluorescence images with exposure times in the range of 2.5–10 s optimized for each condition, capturing up to 4 frames for each condition.

### Image analysis

Digital and analog analysis was carried out using custom scripts written in MATLAB. Briefly, in digital analysis, the center coordinates and radius of each bead in brightfield images (for off-chip analysis) or in darkfield images (for on-chip analysis) were identified using a circular-object detection function (*i.e.*, *imfindcircles*). These coordinates (and the corresponding circles) were then overlaid on fluorescence images, and the maximum pixel intensity was determined for each bead. For the blank condition (zero concentration of analyte), the list of all maximum bead intensities was fitted to a Gaussian distribution. The threshold for distinguishing “on” beads from “off” beads in images from beads exposed to analyte was defined as two standard deviations above the mean of the distribution of bead pixel intensities found in the blank. Consequently, for all non-blank images, all beads with maximum pixel intensities above this threshold value were classified as “on” beads. The fraction of “on” beads *f*_on_ was calculated as the total number of “on” beads divided by the total number of beads counted, and the average number of analyte molecules per bead (AMB) was subsequently calculated as AMB = −ln(1 − *f*_on_). In analog analysis, the average pixel intensity of all fluorescent frames was determined, including both the beads and the background. Subsequently, this value was reported as the fluorescence intensity (in arbitrary units, a.u.).

## Results and discussion

### DABBS assay

The goal of this study was to develop an automated, integrated, microfluidic method for running DABBS assays – that is, d̲igital assays with molecular a̲mplification that result in reporter molecules being b̲ead-b̲ound (but not in a compartment), in a format that allows collection of s̲tatic images. There are three previous reports^13–15^ of DABBS assays that were inspirational, but were not compatible with an automated, integrated microfluidic format. Here, we aimed to develop a microfluidic DABBS assay scheme, and chose to adopt an approach similar to Wu *et al.*,^15^ relying on rolling circle amplification (RCA) to localize the digital signals to beads on a static surface.

As a first step towards this goal, sequences for a biotinylated DNA primer, a DNA template, and a fluorescently labeled DNA probe were adapted from previous reports^28–31^ to allow for on-bead RCA amplification. Briefly, the biotinylated primer is designed to ligate to the DNA template, resulting in the formation of a circular molecular structure. The primer and template then interact with phi29 DNA polymerase to catalyze the formation of an RCA product, a long concatemer of repeated DNA sequences that allows complementary binding of fluorescently labeled DNA probes.

**Fig. 1:**
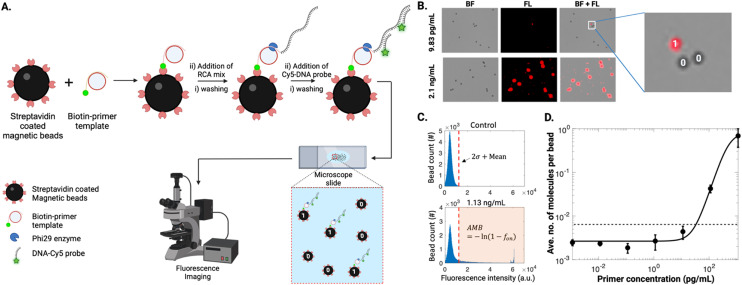
DABBS assay proof-of-concept. (A) Schematic representation of the assay: a biotin-primer template binds to streptavidin-coated beads before initiating RCA, generating a long, immobilized concatemer. Fluorescently-labelled DNA probes hybridize to the concatemer, producing localized fluorescence signals on beads. A droplet containing beads with RCA product (designated as “1”) and without RCA product (designated as “0”) is placed on a microscope slide, and bead counts are obtained through fluorescence imaging. (B) Brightfield (BF), fluorescence (FL), and overlap (BL + FL) microscopy images of beads exposed to low (9.83 pg mL^−1^) and high (2.1 ng mL^−1^) primer concentrations. The zoomed view illustrates one “on” bead (“1”) and two “off” beads (“0”). (C) Plots of normal bead distribution for the control with no primer and 1.13 ng mL^−1^ biotin-primer with a threshold (red dashed line) defined by two standard deviations above the mean value. Beads with intensities below the threshold are considered “off” beads, while those above are “on” beads. The average number of analyte molecules per bead (AMB) is determined by the fraction of “on” *versus* “off” beads. (D) Log–log calibration plot of the average number of molecules per bead *versus* biotin-primer concentration in buffer (black markers) for assays carried out in tubes, fitted with four-parameter logistic (4PL) curve (black trace), with the limit of detection (LOD) (dashed black line) corresponding to three standard deviations above the mean of the control. Error bars represent mean ± standard error for *n* = 3 per condition.

A proof-of-concept DABBS assay procedure was then developed, as illustrated in Fig. 1A. Briefly, a suspension of streptavidin-coated beads was exposed sequentially to solutions of biotinylated DNA primer, DNA template, phi29 DNA polymerase, dNTPs, and fluorescently labelled DNA probes, interspersed with multiple bead pelleting, wash, resuspension, and incubation steps. After the assay, the final suspension of beads was imaged by microscopy to collect brightfield and fluorescence images to count the “on” and “off” beads. As shown in Fig. 1B, at high concentrations of biotinylated DNA primer (the “analyte” in this proof-of-concept example), nearly all beads appeared to be completely covered with RCA products, as indicated by the presence of fluorescence signals across the entire 2-D projection of the beads. However, at sufficiently low concentrations of analyte, the signals were reduced such that only a fraction of the beads appears with high fluorescence intensity. Using a custom-written MATLAB script, we measured the maximum intensity from individual beads (Fig. 1C). From control experiments, we established a threshold (2× standard deviation + mean) for defining “off” (*i.e.*, 0) and “on” (*i.e.*, 1) beads for calculating the fraction of “on” beads, *f*_on_, that was used to determine the average number of analyte molecules per bead, AMB [note that as described previously,^15^ AMB is analogous to the average number of enzymes per bead (AEB) that is used in conventional Simoa measurements]. We then fitted the measured data using a 4-parameter logistic model to obtain the calibration curve for biotin-DNA primer (Fig. 1D). Using three standard deviations above the mean of blank, we obtained the limit of detection (LOD) of 38.35 pg mL^−1^ (Fig. 1D).

In practice, the compartmentalization-free DABBS assay format introduced here shares many characteristics with the three that have been reported previously.^13–15^ Most importantly, as with previous reports,^13–15^ there were no “compartments” needed for the new assay, a feature that greatly simplifies the detection and analysis. One innovation and critical difference for the DABBS technique introduced here is that it was designed for the beads to be analyzed in a format that is compatible with microfluidics: a simple dispersion in aqueous medium sandwiched between glass substrates. This stands in contrast to the DABBS methods reported previously that required beads be suspended in a hydrogel matrix in an open chamber,^13^ positioned in an open electrochemical cell,^14^ or drop-cast (dry) on an open surface.^15^

In summary, the method illustrated in Fig. 1 demonstrates proof-of-concept for performing a DABBS assay in a microfluidics-compatible format. Our next aim was to modify the assay to be useful for detecting protein targets. For this, we chose the SARS-CoV-2 spike protein as a model analyte, as described below.

### DABBS assay for protein detection

Transitioning from the proof-of-concept assay illustrated in Fig. 1 to a digital assay for protein analytes requires incorporation of protein-recognition elements. In digital immunoassays, protein capture and detection has been traditionally accomplished by using a pair of highly specific immunoglobulin-based conventional antibodies (mAbs) produced in mammalian hosts. Here, we chose to use nanobodies derived from camelid heavy chain-only antibodies^22^ (V_H_Hs), because of the ease with which these reagents can be produced, modified and scaled for production.^33^ A pair of capture and detection nanobodies was selected to report the concentration of recombinant SARS-CoV-2 spike protein. In the context of viral antigen tests,^34^ spike protein is more sensitive to viral mutation than other antigens,^35^ which can be an advantage (for discriminating between variants) or a disadvantage (not universal). Regardless, it served as a useful test-case for the compartmentalization-free DABBS assay development described here.

**Fig. 2:**
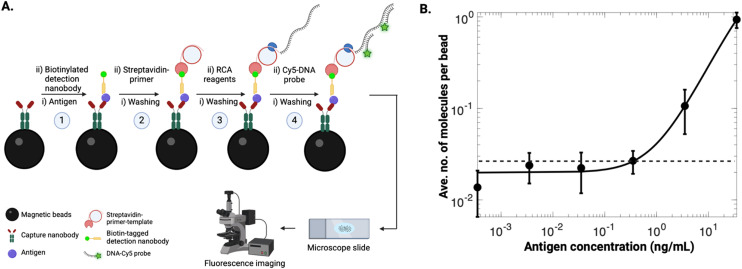
DABBS assay for recombinant SARS-CoV-2 spike protein. (A) Schematic representation of the assay: antigen bound to capture nanobody-coated beads that was sequentially bound to a biotinylated detection nanobody and SPT. RCA was initiated, and the resulting concatemers were labeled with the fluorescently-tagged DNA probes, and the beads were imaged using a fluorescence microscope. (B) Log–log calibration plot of the average number of molecules per bead *versus* the concentration of SARS-CoV-2 spike protein in buffer (black markers) for assays carried out in tubes, fitted with 4PL curve (black trace), with LOD (black dashed line) corresponding to three standard deviations above the control level. Error bars represent mean ± standard error for *n* = 3 per condition.

A schematic of the DABBS protein detection assay is shown in Fig. 2A. Beads were conjugated with the capture nanobody and then incubated with the antigen and biotinylated detection nanobody to form a sandwich immunoassay. Then, streptavidin–primer–template (SPT) was added to the immunocomplexes, and subsequent amplification by RCA allowed for binding of fluorescently labeled DNA probes. Reagent concentrations for the assay were carefully optimized (Note S1, Fig. S2 and S3†), resulting in an LOD of 0.36 ng mL^−1^ (equivalent to 36 pg of antigen in 100 μL sample volume used in the assay). This LOD was more than an order of magnitude lower than those of commercial test kits for SARS-CoV-2 spike protein [6.3 ng mL^−1^ (ref. 36), 23 ng mL^−1^ (ref. 37), and 31 ng mL^−1^ (ref. 38)], highlighting the potential benefits of using this type of digital assay relative to standard analog assays.

### DABBS assay comparisons

With any new assay, it is necessary to compare and validate its performance to that of comparable tests. However, because of the vast differences in analytes (not to mention other assay parameters), it is not possible to compare the performance of the new DABBS assay for SARS-CoV-2 spike protein to the three DABBS assays described previously [which were developed for IL-6 (ref. 13), IgG (ref. 14), IL-1 (ref. 15), and IL-10 (ref. 15)]. Thus, we developed our own comparator assays for the same analyte, to provide suitable context for DABBS assay introduced here.

**Fig. 3:**
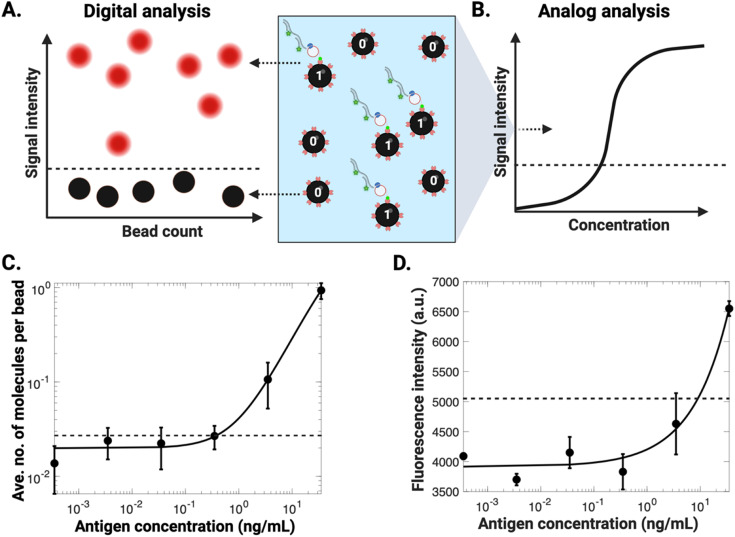
Analog *versus* digital detection schemes. (A and B) Schematic representations of the DABBS assay described here, evaluated with (A) digital or (B) analog detection schemes. The bead suspension is shown in the middle panel with the carrier solution illustrated in blue. In the digital approach (A), “on” beads (shown in red) and “off” beads (shown in black) were counted, whereas in the analog analysis (B), an average intensity from the bead suspension was captured. (C and D) Calibration plots of signal *versus* the concentration of recombinant SARS-CoV-2 spike protein in buffer analyzed by (C) digital or (D) analog detection schemes for assays carried out in tubes (note that Fig. 3C is a repeat of Fig. 2B for ease of comparison). Data are shown as black markers and are fitted with 4PL curves (black traces), with LOD (black dashed line) corresponding to three standard deviations above the control level. Error bars represent mean ± standard error for *n* = 3 per condition.

An obvious comparator for the new DABBS assay was to simply apply an ‘analog’ detection scheme to the same data that was used to collect the DABBS signal. This concept is represented in Fig. 3A and B – the same experiments can be used to generate both ‘digital’ and ‘analog’ (average) signals, either by counting the number of “on” and “off” beads (Fig. 3A) or by measuring the average signal across the entire images, which includes both beads and the liquid in the sample (Fig. 3B). The analyses for the two schemes are shown in Fig. 3C and D. As indicated, the digital assay (Fig. 3C) is clearly superior to the analog assay (Fig. 3D), with an improvement in LOD of more than an order of magnitude.

The digital *versus* analog comparison illustrated in Fig. 3 makes the case for digital detection, but we decided to explore a series of related assays for the same analyte, to evaluate other aspects of the DABBS paradigm. LODs for five assays (including DABBS and four comparators) are shown in Table 1. The analog version of the DABBS assay (Fig. 3B and D) is listed in the table as comparator 1. A digital and an analog version of the same assay without molecular amplification (Note S2, Fig. S4†) is listed in the table as comparators 2 and 3. An analog, colorimetric version of the assay with molecular amplification (Note S3, Fig. S5†) is listed in the table as comparator 4. From this comparison, it is clear that digital is preferred over analog, amplification is preferred over non-amplification, and fluorometric is more sensitive than colorimetric. These results were expected, but it is useful to quantify the differences in assay formats to help determine the analytical value of each approach. Our final goal was to port the DABBS scheme to digital microfluidic format.

**Table 1:** Comparison of DABBS to comparator assays for detection of SARS-CoV-2 spike protein for assays carried out in tubes

	DABBS	Comparator 1	Comparator 2	Comparator 3	Comparator 4
Detection mode (reporter)	Fluorescence (Cy5)	Fluorescence (Cy5)	Fluorescence (Cy5)	Fluorescence (Cy5)	Absorbance (TMB)
Amplification (method)	Yes (RCA)	Yes (RCA)	No	No	Yes (HRP)
Detector	Microscope	Microscope	Microscope	Microscope	Spectro-photometer
Analysis mode	Digital	Analog	Digital	Analog	Analog
LOD	0.36 ng mL^−1^	9.27 ng mL^−1^	56.48 ng mL^−1^	244.11 ng mL^−1^	20.91 ng mL^−1^

### Microfluidic DABBS assay

**Fig. 4:**
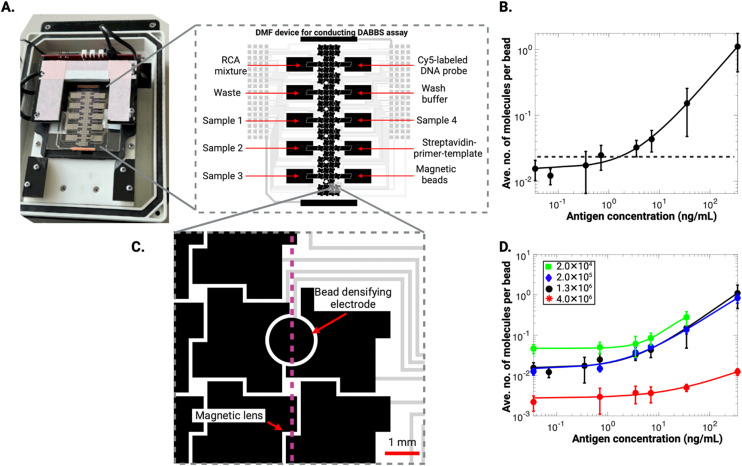
Microfluidic DABBS assay. (A) Photograph (left) of the DMF assay chip used for bead processing connected to a custom-made actuation box featuring a pogo pin-electrode pad interface for programming electrode actuations, along with an adjustable magnetic lens for bead pelleting/densifying, and schematic (right) that indicates the array of electrodes (black), including reservoirs dedicated for RCA mixture, waste, and samples 1–3 (on the left) and labelled DNA probe, wash buffer/negative control, sample 4, SPT solution, and magnetic bead suspension (on the right). The electrode pads and wires are shown in gray. The five different reaction zones are indicated as white round spots on the array of electrodes. (B) Log–log calibration plot of the average number of molecules per bead *versus* the concentration of recombinant SARS-CoV-2 spike protein in buffer (black markers) for DABBS assays with 1.3 × 10^6^ beads carried out on-chip with off-chip detection, fitted with 4PL curve (black trace), with LOD (black dashed line) of 1.64 ng mL^−1^ corresponding to three standard deviations above the control level. (C) Schematic of a magnified region of a modified DMF bottom plate featuring a round densifying electrode positioned over the magnetic lens (dashed magenta line). (D) Log–log calibration curves of the average number of molecules per bead *versus* the concentration of recombinant SARS-CoV-2 spike protein in buffer (markers) for DABBS assays carried out on-chip with off-chip detection, fitted with 4PL curves generated using different bead numbers: 2.0 × 10^4^ (green), 2.0 × 10^5^ (blue), 1.3 × 10^6^ (black, replotted from panel B) and 4.0 × 10^6^ (red) beads. Error bars in (B) and (D) represent mean ± standard errors for *n* = 3 per condition.

After developing the DABBS assay and comparing it to other formats, we re-engineered it to be performed “on chip” (*i.e.*, automated in a DMF platform), a process that required revisions to the sample/reagent makeup and volumes (see Experimental section for details). Fig. 4A shows the DMF system, consisting of a device bearing an array of electrodes and a custom-made actuation box equipped with droplet control electronics and an automated, mechanical magnetic lens. A custom 19-step program was developed in the open-source MicroDrop software environment,^26^ including application of electric fields to control droplet position and the position of a magnetic lens.^39^ The latter, positioned a few centimeters below the DMF device, was activated (raising it close to the device) to immobilize particles on the device surface for reagent exchange, and was deactivated (lowering it away from the device) to allow for particles to be dispersed (see Video S1†). In initial experiments, the microfluidic DABBS assay was evaluated quantitatively by collecting the beads from the device after the 19-step procedure and transferring them to an epifluorescence microscope for imaging. A calibration curve for the assay is shown in Fig. 4B, with an LOD of 1.64 ng mL^−1^, corresponding to 3.28 pg of analyte in the 2 μL sample volume used here. In terms of concentration detection limits, this LOD is lower than the LODs reported for most commercial kits, as discussed above. And in terms of absolute detection limits, this LOD is lower than the one observed for the off-chip DABBS assay described above. We speculate that this improvement in assay performance may be a result of the improved binding and washing efficiencies in the smaller volumes handled in the microfluidic format relative to the larger volumes handled in tubes, but additional work is needed to test this explanation.

The microfluidic assay data described above (Fig. 4B) were generated using the same number of beads (1.3 × 10^6^) that was used for the manual assays that were tested in tubes. In fact, bead counts in this range are standard in most digital microfluidic bead-processing studies, as large numbers of beads are required for effective bead-pelleting for fluid exchange. We recently demonstrated^40^ an alternative strategy relying on the use of specially designed bead densification electrodes that allows for the reliable manipulation and recovery of much smaller numbers of magnetic beads in digital microfluidics. This approach is a useful innovation, as Simoa assays are known^41,42^ to have increased sensitivity for reduced numbers of beads by increasing the ratio of target molecules to beads and thereby the slope of the dose–response curve of the assay. We decided to apply this densification electrode strategy here, to be able to use small numbers of beads and to optimize the performance of the microfluidic DABBS assay. A modified device design featuring bead densifying electrodes is shown in Fig. 4C. A range of bead numbers (2.0 × 10^4^–4.0 × 10^6^) was explored, and calibration curves for the DABBS assay for four different conditions are displayed in Fig. 4D. As shown, the lowest LOD observed was 0.67 ng mL^−1^ in experiments using 2.0 × 10^5^ beads, while higher LODs of 1.64 ng mL^−1^ and 328.61 ng mL^−1^ were observed for 1.3 × 10^6^ and 4.0 × 10^6^ beads, respectively. The increased LOD for 4.0 × 10^6^ beads can likely be attributed to the lower assay slope for the higher bead counts.^42,43^ Similarly, the higher LOD for 2.0 × 10^4^ beads is likely due to a lower assay slope, which results from the use of fewer beads. The use of fewer beads leads to fewer capture nanobodies, in turn, lower capture efficiency due to reduced antibody concentration in the antibody–antigen reaction.^42,43^ The best performing condition (2.0 × 10^5^ beads) consistently resulted in the lowest LOD, representing an optimal bead number in terms of the trade-off between the number of protein molecules and beads, as well as the kinetics of capture driven by the availability of capture nanobodies. As shown, the signal-concentration relationship was similar for 2.0 × 10^5^ beads and 1.3 × 10^6^ beads; the difference that led to a smaller LOD was the reduced standard errors observed for the case of 2.0 × 10^5^ beads. This condition was used for all subsequent experiments.

In summary, Fig. 4 outlines, to our knowledge, the first report of a microfluidic DABBS assay. Note that digital immunoassays have been demonstrated previously on DMF devices,^44,45^ but they required integrated, microfabricated microwell arrays for bead compartmentalization (*i.e.*, requiring substantially more complex devices and methods than the DABBS format). Our final goal was to apply the technique to the evaluation of analyte spiked in ‘real’ sample matrix in a format consistent with future applicability as a diagnostic tool outside of the laboratory (described below).

### Microfluidic DABBS assays in saliva

**Fig. 5:**
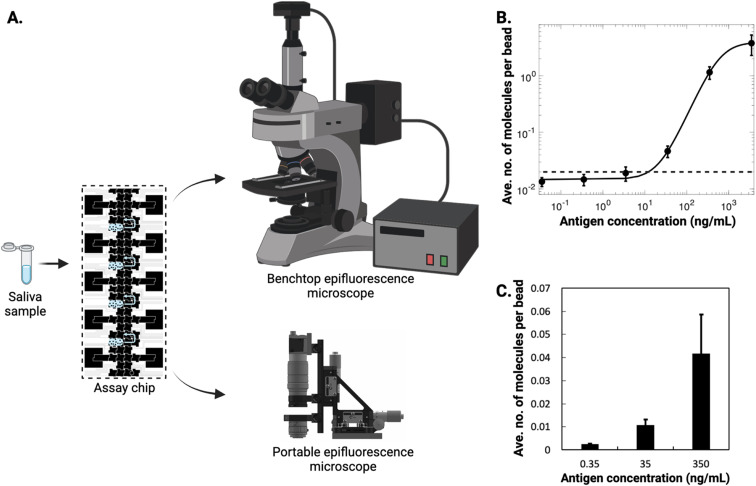
Microfluidic DABBS assay in saliva. (A) Schematic of the microfluidic DABBS assay in saliva samples (left), with imaging off-chip (right) *via* benchtop epifluorescence microscope (schematic) or on-chip using a portable epifluorescence microscope (3-D rendered design). (B) Log–log calibration plot of the average number of molecules per bead *versus* the concentration of recombinant SARS-CoV-2 spike protein in saliva (black markers) for DABBS assays with 2.0 × 10^5^ beads carried out on-chip with off-chip detection, fitted with 4PL curve (black trace), with LOD (black dashed line) of 15.72 ng mL^−1^ corresponding to three standard deviations above the control level. (C) Plot of the average number of molecules per bead *versus* the concentration of SARS-CoV-2 spike protein in saliva (black bars) for DABBS assays with 2.0 × 10^5^ beads carried out on-chip using a low-cost imager.^32^ Error bars in (B) and (C) represent mean ± standard error for *n* ≥ 3 per condition.

We next evaluated the performance of the new assay using saliva, a sample type that has been proposed for use in diagnosing viral infections, including SARS-CoV-2.^46^ Saliva is particularly challenging to work with due to its viscosity and particulate content, so was a useful specimen for proof-of-principle work. To test the performance of the DABBS assay in saliva, known amounts of recombinant SARS-CoV-2 spike protein were spiked into pooled saliva collected from human patients. Undiluted samples of spiked saliva (without any further sample processing) were loaded into digital microfluidic devices (Fig. 5A) to evaluate the assay's performance in this matrix (Fig. 5B). As shown, the assay had an LOD of 15.72 ng mL^−1^, corresponding to 31.44 pg of antigen in the 2 μL volume of saliva used. This LOD is higher than the one measured in buffer (Fig. 4). The phenomenon of increased detection limits for immunoassays when using complex samples like saliva (relative to controlled buffer) is well known^47^ and is commonly attributed to nonspecific interference from salivary proteins. However, even with this reduction in performance, the LOD for the microfluidic DABBS assay in saliva is excellent in the context of alternative tests, comparing favorably to recent reports of assays for this antigen in this matrix – *e.g.*, LODs of 1.45 ng (ref. 48) in 50 μL saliva and 5.7 ng (ref. 49) in 300 μL saliva. In future applications requiring additional analytical sensitivity in complex matrices, new methods^40^ allowing for on-chip pre-concentration from large sample volumes may be useful to consider.

The final step to making the system compatible with portable applications is to reduce the size and cost of the imager, as all the data referenced above was collected off-chip using a standard commercial (benchtop) epifluorescence microscope. We, therefore, evaluated the use of an inexpensive portable microscope^32^ to image DABBS on a DMF device. A series of undiluted saliva samples were spiked with antigen at different concentrations, loaded into DMF devices, and evaluated on-chip, allowing for detection at similarly low concentrations (Fig. 5C). In future work, the portable detector might be miniaturized even further,^50–53^ but these results are promising, demonstrating the potential for moving highly sensitive digital detection out of the lab, and into settings closer to the point of need.

## Conclusions

We have developed a compartmentalization-free digital detection assay utilizing rolling circle amplification for enhanced sensitivity. We optimized and integrated the new assay into an automated digital microfluidic platform for the detection of SARS-CoV-2 spike protein, enabling an LOD of 1.64 ng mL^−1^ (3.28 pg in 2 μL sample volume) in buffer and 15.72 ng mL^−1^ (31.44 pg in 2 μL sample volume) in saliva. Finally, proof-of-concept data were collected using a portable imaging system, demonstrating the potential of the platform to become a fully functional point-of-care device. In summary, the combination of digital immunoassays with the DMF platform sets the stage for a wide range of applications requiring high sensitivity assays combined with portability for applications outside of the laboratory.

## Author contributions

N. L., N. S., and A. S. contributed equally to this study. A. S. developed the DABBS assay and wrote the image analysis algorithms. N. L. and N. S. developed the DABBS assay for protein analytes. N. L., N. S., and A. S. designed and conducted the experiments and analyzed data. N. L., N. S., A. S., and A. R. W. wrote the manuscript. R. M., R. E. M., A. D. W., and C. W. K. developed and used the portable imaging platform. M. A. R. and J. T. developed and modified the nanobodies against SARS-CoV-2 spike protein. S. D. conducted initial background work. R. P. S. d. C. and M. D. C. conceptualized the idea. D. C. D., N. P., and A. R. W. secured funding for the study and provided supervision. All authors contributed to the discussion of the results and the conceptualization of the study.

## Conflicts of interest

M. A. R. and J. T. declare the following competing interests. National Research Council Canada has filed a patent (PCT/IB2022/053756, “Antibodies that bind SARS-CoV-2 spike protein”) that includes the two V_H_Hs described here with M. A. R. and J. T. named as inventors. R. M., R. E. M, C. W. K., A. D. W., and D. C. D. are employees of Quanterix Corporation that develops and markets products for digital detection of proteins. All other authors declare no competing interests.

## Supplementary Material

LC-025-D5LC00103J-s001

LC-025-D5LC00103J-s002

## Data Availability

The raw data associated with this manuscript is available from the corresponding author upon request.
