# Supplementary material for: A compartmentalization-free microfluidic digital assay for detecting picogram levels of protein analytes
Source: Lab Chip. 2025 May 12;25(12):2862–73. doi: 10.1039/d5lc00103j (PMC12067047; doi:10.1039/d5lc00103j)
Supplement: LC-025-D5LC00103J-s001 [file LC-025-D5LC00103J-s001.pdf]

Supplementary Information (SI) for Lab on a Chip.
This journal is © The Royal Society of Chemistry 2025

Supplementary Information for **A Compartmentalization-Free Microfluidic Digital Assay for Detecting Picogram Levels of Protein Analytes**

Nguyen H. Le,[†ab] N. Sathishkumar,[†ab] Alinaghi Salari,[†ab] Ryan Manning,[c] Raymond E. Meyer,[c] Cheuk W. Kan,[c] Alexander D. Wiener,[c] Martin A. Rossotti,[d] Sheldon Decombe,[ab] Richard P. S. de Campos,[abe] M. Dean Chamberlain,[abfg] Jamshid Tanha,[d,h] Nira R. Pollock,[i] David C. Duffy,[c]

a. Department of Chemistry, University of Toronto, 80 St. George Street, Toronto, ON M5S 3H6, Canada.
b. Terrence Donnelly Centre for Cellular and Biomolecular Research, University of Toronto, 160 College Street, Toronto, ON M5S 3E1, Canada.
c. Quanterix Corporation, 900 Middlesex Turnpike, Billerica, MA 01821, USA.
d. Human Health Therapeutics Research Centre, Life Sciences Division, National Research Council Canada, Ottawa, ON K1A 0R6, Canada.
e. Current address: Quantum and Nanotechnologies Research Centre, National Research Council Canada, Edmonton, AB T6G 2M9, Canada.
f. Institute of Biomedical Engineering, University of Toronto, 164 College Street, Toronto, ON M5S 3E2, Canada.
g. Current address: Department of Discovery and Translation Research and Department of Oncology, University of Saskatchewan, 107 Wiggins Road, Saskatoon, SK S7N 5E5, Canada.
h. Department of Biochemistry, Microbiology and Immunology, Faculty of Medicine, University of Ottawa, 451 Smyth Road, Ottawa, ON K1H 8M5, Canada.
i. Department of Laboratory Medicine, Boston Children's Hospital, 300 Longwood Avenue, Boston, MA 02115, USA.

† Equal contribution

and Aaron R. Wheeler[*abf]

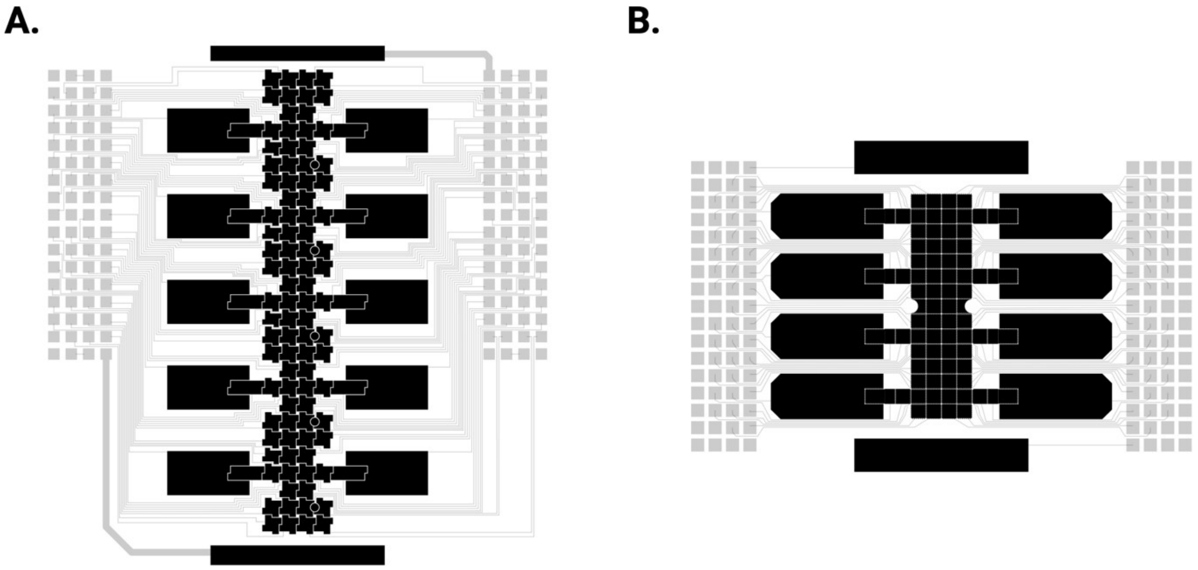

Figure S1. CAD schematics of the two DMF chip designs used in this study: (A) the "Assay" chip and (B) the "Image" chip. The "Assay" chip was used for running the on-chip DABBS, while the "Image" chip was used for conducting imaging with the portable imager.

Note S1: Optimization of detection nanobody and streptavidin-primer conjugate concentrations for DABBS assays for SARS-CoV 2 spike protein. DABBS assays were carried out off-chip as described in the main text, using varying concentrations of detection nanobody, and streptavidin-primer-template (SPT). As shown in Fig. S2, the highest concentration of detection nanobody (20 µg/mL) resulted in the highest signal; however, high background was observed in the blank for this condition (data not shown). Thus, 1 µg/mL detection nanobody was used for the remainder of the experiments. As shown in Fig. S3, the highest concentration (17.2 µg/mL) of SPT showed the highest average number of molecules per bead value and thus was used for the remainder of the experiments.

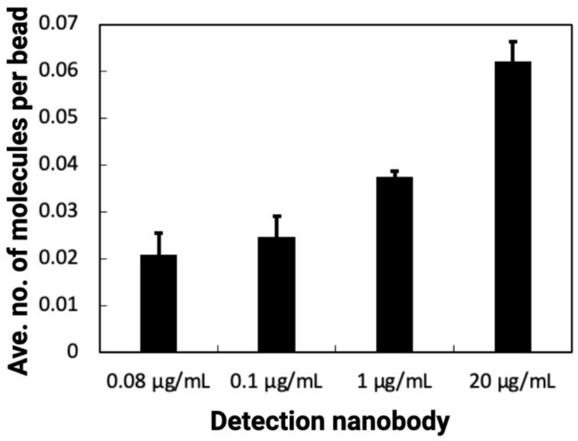

Figure S2. Plot of average number of molecules per bead for DABBS assays carried out in tubes for 81.7 µg/mL of antigen and 1.7 µg/mL of streptavidin-primer-template (SPT) as a function of detection nanobody concentration. Error bars represent mean ± standard error for n ≥ 2 per condition.

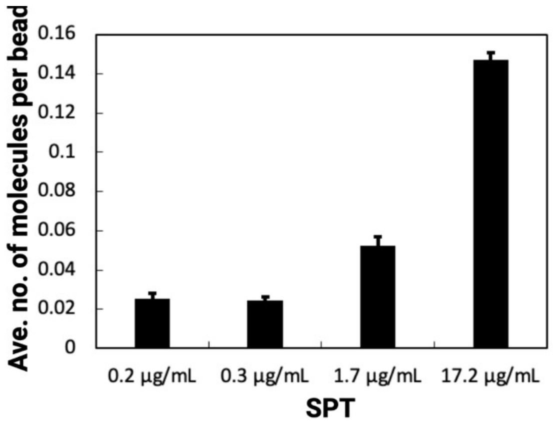

Figure S3. Plot of average number of molecules per bead for DABBS assays carried out in tubes for 81.7 µg/mL of antigen with 1 µg/mL detection nanobody as a function of SPT concentration. Error bars represent mean ± standard errors for n ≥ 2 per condition.

Note S2: Off-chip digital and analog fluorometric assays without amplification for SARS-CoV-2 spike protein. A non-amplified fluorometric assay was developed relying on binding of streptavidin-Cy5. Briefly, a suspension of capture nanobody-coated beads, and the solutions of antigen, and biotinylated detection nanobody were mixed and then washed in SuperBlock™ PBS as described in the 'off-chip DABBS procedure' section in the main text. A 50-μL aliquot of streptavidin-Cy5 (4.4 μg/mL) was added to the mixture that was rotated for 30 min at room temperature. The beads were then washed six times in SuperBlock™ PBS and transferred to a microscope slide for imaging as described in the main text. A schematic of the assay is shown in Fig. S4A, and calibration curves evaluated under digital and analog schemes are shown in Fig. S4B and Fig. S4C, respectively.

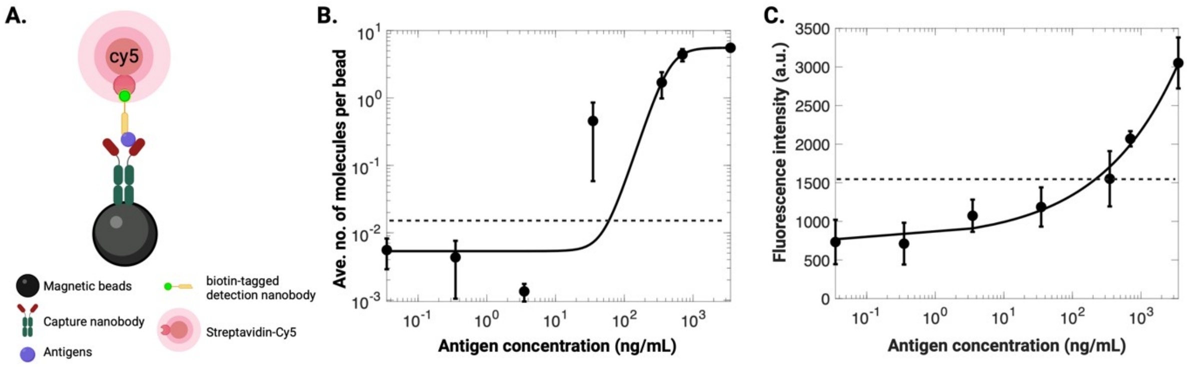

Figure S4. (A) Schematic of the fluorometric assay for SARS-CoV-2 spike protein without amplification based on streptavidin-Cy5 labeling. (B, C) Log-log/semi-log calibration plots of average number of molecules per bead versus antigen concentration in buffer (black markers) for assays carried out in tubes, fitted with four parameter logistic curves (black traces) for analysis by (B) digital or (C) analog detection schemes. Error bars represent mean ± standard error for n = 3 per condition. Limits of detection (dashed black lines) corresponding to three

standard deviations above the mean of the control were 56.48 ng/mL and 244.11 ng/mL for analog and digital analysis, respectively.

Note S3: Off-chip analog colorimetric assay with amplification for SARS-CoV-2 spike protein. An analog colorimetric immunoassay was developed in which a streptavidin-horseradish peroxidase (strep-HRP) conjugate was used to generate signal in tubes. Briefly, the suspension of capture nanobody-coated beads, and the solutions of antigen and biotinylated detection nanobody were mixed and then washed in SuperBlock™ PBS as described in the 'DABBS for SARS-CoV-2 spike protein assays' section in the main text. A 50-µL aliquot of strep-HRP (diluted 1:10,000, as recommended by the manufacturer) was introduced and incubated for 30 min at room temperature. The beads were pelleted, and the supernatant was removed from the tubes using a pipette. The beads were then incubated with 5 µL of one-step TMB (3,3',5,5'-tetramethylbenzidine) substrate (Sigma Aldrich, ON, Canada) for 5 min at room temperature. The reaction was quenched by adding 0.5 µL 1 M H_2SO_4 to each tube and the absorbance at 450 nm was measured immediately by adding 1 µL to the Nanodrop One Spectrophotometer (Thermo Fischer, ON, Canada). A schematic is shown in Fig. S5A, and a calibration curve (Fig. S5B) was generated by measuring the optical density (OD450) using the Nanodrop.

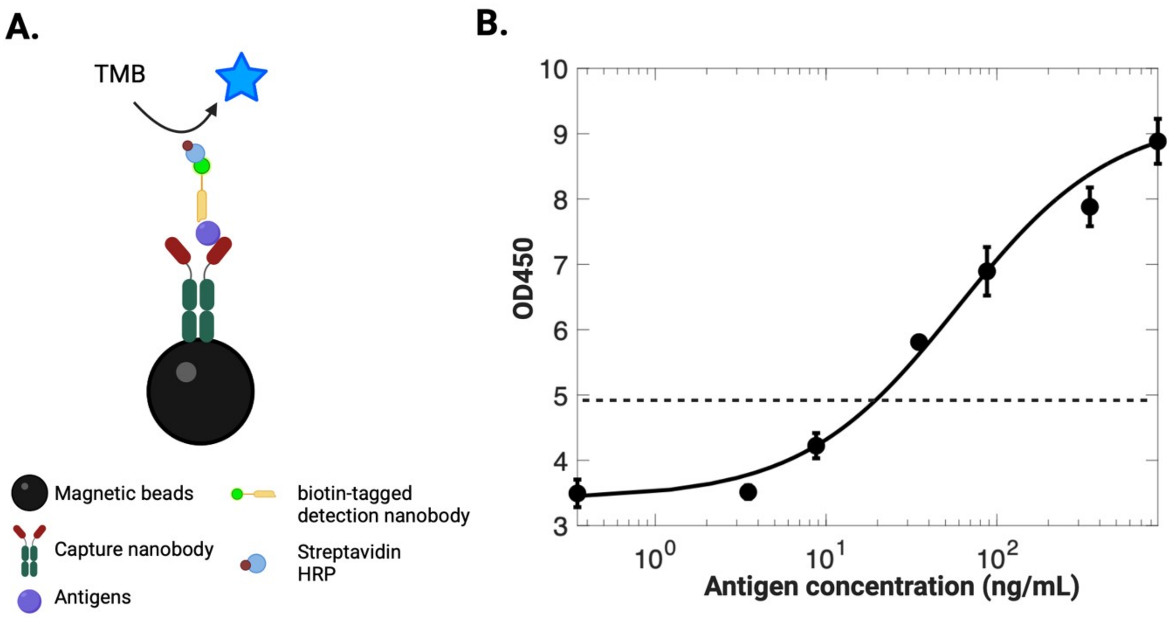

Figure S5. (A) Schematic of the analog colorimetric assay for SARS-CoV-2 spike protein, relying on strep-HRP activity. (B) Semi-log calibration plot of antigen concentration in buffer (black markers) for assays carried out in tubes, fitted with four parameter logistic curve (black trace). Error bars represent mean $\pm$ standard errors for n = 3 per condition. Limit of detection (dashed black line) corresponding to three standard deviations above the mean of the control was 20.9 ng/mL.

Video S1: The video (8× speed) demonstrates multiple washing steps for three samples in parallel on a type-A DMF chip. During each wash, the beads in each sample were first dispersed in a droplet of wash-buffer, with the magnetic lens disengaged. The magnetic lens was then engaged to trap the beads in a 200-nL droplet, while the remainder of the fluid was moved to waste. The samples in the left and middle contained 2.0×10^4 beads (which form a pellet that is difficult to see at this magnification), while the sample at the right contained 2.0×10^5 beads (which form an observable pellet). The sample on the left was spiked with soluble red dye (not retained by the beads) to illustrate the washing process.
